# Using Web-Based Pin-Drop Maps to Capture Activity Spaces Among Young Adults Who Use Drugs in Rural Areas: Cross-Sectional Survey

**DOI:** 10.2196/13593

**Published:** 2019-10-18

**Authors:** Hannah Luke Fenimore Cooper, Natalie D Crawford, Regine Haardörfer, Nadya Prood, Carla Jones-Harrell, Umedjon Ibragimov, April M Ballard, April M Young

**Affiliations:** 1 Rollins School of Public Health Emory University Atlanta, GA United States; 2 College of Public Health University of Kentucky Lexington, KY United States

**Keywords:** rural, substance use disorder, Web-based data collection, geospatial methods, risk environment, activity spaces

## Abstract

**Background:**

Epicenters of harmful drug use are expanding to US rural areas, with rural young adults bearing a disproportionate burden. A large body of work suggests that place characteristics (eg, spatial access to health services) shape vulnerability to drug-related harms among urban residents. Research on the role of place characteristics in shaping these harms among rural residents is nascent, as are methods of gathering place-based data.

**Objective:**

We (1) analyzed whether young rural adults who used drugs answered self-administered Web-based mapping items about locations where they engaged in risk behaviors and (2) determined the precision of mapped locations.

**Methods:**

Eligible individuals had to report recently using opioids to get high; be aged between 18 and 35 years; and live in the 5-county rural Appalachian Kentucky study area. We used targeted outreach and peer-referral methods to recruit participants. The survey asked participants to drop a pin in interactive maps to mark where they completed the survey, and where they had slept most; used drugs most; and had sex most in the past 6 months. Precision was assessed by (1) determining whether mapped locations were within 100 m of a structure and (2) calculating the Euclidean distance between the pin-drop home location and the street address where participants reported sleeping most often. Measures of central tendency and dispersion were calculated for all variables; distributions of missingness for mapping items and for the Euclidean distance variable were explored across participant characteristics.

**Results:**

Of the 151 participants, 88.7% (134/151) completed all mapping items, and ≥92.1% (>139/151) dropped a pin at each of the 4 locations queried. Missingness did not vary across most participant characteristics, except that lower percentages of full-time workers and peer-recruited participants mapped some locations. Two-thirds of the pin-drop sex and drug use locations were less than 100 m from a structure, as were 92.1% (139/151) of pin-drop home locations. The median distance between the pin-drop and street-address home locations was 2.0 miles (25th percentile=0.8 miles; 75th percentile=5.5 miles); distances were shorter for high-school graduates, staff-recruited participants, and participants reporting no technical difficulties completing the survey.

**Conclusions:**

Missingness for mapping items was low and unlikely to introduce bias, given that it varied across few participant characteristics. Precision results were mixed. In a rural study area of 1378 square miles, most pin-drop home addresses were near a structure; it is unsurprising that fewer drug and sex locations were near structures because most participants reported engaging in these activities outside at times. The error in pin-drop home locations, however, might be too large for some purposes. We offer several recommendations to strengthen future research, including gathering metadata on the extent to which participants zoom in on each map and recruiting participants via trusted staff.

## Introduction

Epicenters of harmful drug use in the United States are shifting from cities to rural areas; rural young adults bear the brunt of these epidemiologic transitions [1]. Since the mid-1990s, rates of nonmedical prescription opioid use and heroin use have surged among young adults in rural areas. As a result, young adults in rural areas are now at the forefront of the national hepatitis C virus (HCV) epidemic, and their children bear a disproportionate burden of neonatal abstinence syndrome [2,3].

A large body of theoretical and empirical work suggests that characteristics of the *activity spaces* of people who use drugs shape their drug use patterns, drug-related harms, and health service use. Activity spaces are defined as “local areas within which people move or travel in the course of their daily activities” [4]. The Risk Environment Model, Social Ecological Model, Andersen’s Behavioral Model of Health Care Utilization, and other theoretical frameworks posit that characteristics of places (eg, census tract poverty rates and income inequality in states) powerfully affect health behavior and health service use [5-7]. Evidence supports these propositions among those living in cities. For example, urban people who use drugs are more likely to use health care services that are closer to where they live, and those living in areas with greater densities of dilapidated buildings are more likely to overdose [8,9]. Documenting the characteristics of the activity spaces of young adults who use drugs can elucidate why some people who use drugs are more likely to engage in risk behavior than others and can strengthen planning efforts to ensure spatial access drug-related [3] health services (eg, syringe service programs, drug treatment programs).

The methods to measure activity spaces were, however, developed in urban environments and are predicated on assumptions that may not hold for rural populations or populations engaging in illegal behavior, including rural young adults who use drugs. For example, studies of activity spaces often require that participants travel to the study storefront, either to take part in an interviewer-administered survey about activity spaces or to retrieve a global positioning system (GPS) unit (which later needs to be returned). Traveling to a storefront may, however, be difficult in rural areas, where travel distances are longer on average than they are in cities; public transportation is rare; and low-income residents (including young adults who use drugs) often lack cars [10-12]. Moreover, methods that require participants to travel with a GPS unit that records their movements may not be acceptable to people who use drugs who engage in illegal behavior. Survey-based methods of capturing activity spaces may also fail. These surveys tend to query specific addresses where an activity occurs, or the nearest intersection. In rural areas, however, activities may occur in places that have no street address (eg, in a forest, by a lake), and intersections are relatively rare.

Interactive digital maps embedded in a Web-based survey might, however, prove a valid alternative method of capturing activity spaces of rural young adults who use drugs. Young adults in rural areas report high rates of internet use [13]. Web-based interactive maps bypass the need to travel to a storefront or carry a GPS unit and would not require that participants report street addresses or intersections. This method has proved feasible for men who have sex with men and generates relatively precise geospatial data [14]. This method’s feasibility for young adults who use drugs is, however, unknown, as is the validity of the resulting geospatial data. Technical difficulties navigating digital interactive maps and hesitation to disclose the locations of illegal or stigmatizing behaviors could impact data quality.

Here, we test the *feasibility* of using self-administered interactive digital maps embedded in a Web-based survey to capture risk-related activity spaces of rural, young adults who use drugs, and the *precision* of the resulting geospatial data. We define *risk-related* activity spaces as the *local areas within which people move or travel in the course of their daily activities while engaging in drug-related and sexual risk behaviors*. Participants were invited to *drop pins* in these digital maps to mark particular locations (eg, where they have slept most and where they have misused prescription opioids most). Specifically, our analyses were designed to explore the following questions:

1. Do participants report technical difficulties while completing Web-based mapping items?
2. Do participants answer mapping questions about their risk-related activity spaces, and does item completion vary by participant characteristics, including sociodemographic characteristics and drug-related behaviors?
3. How precise are pin-drop mapping data about the locations where participants live and engage in risk-related behaviors?

## Methods

### Survey Overview

Questions about activity spaces, sociodemographic characteristics, and technical difficulties were queried as part of a self-administered Web-based survey about risk of HIV and HCV infection and overdose. This survey was administered between August 27, 2017 and July 31, 2018 to young adults who use drugs living in rural Appalachian Eastern Kentucky, a region with high rates of HCV and overdoses [2,15]. The Web-based survey was programmed in SurveyGizmo [16].

### Eligibility and Recruitment

To be eligible to take part in the self-administered Web-based survey, individuals had to be aged between 18 and 35 years; currently live in 1 of the 5 rural Appalachian Kentucky counties studied here; and report recent (past 30 days) use of an opioid to get high; opioids included heroin, prescription pain pills (eg, Percocet), and medication-assisted therapies (eg, buprenorphine).

Participants were recruited using targeted outreach and Web-based peer-referral methods. Targeted outreach strategies included (1) holding neighborhood cookouts in areas with high rates of overdoses and other drug-related harms; (2) disseminating information about the study through community partners and through staff working in another local study of adults who use drugs (the “CARE2HOPE” study); and (3) posting flyers in places where young adults who use drugs might spend time (eg, gas stations, health departments, and social service offices). Regardless of recruitment method, participants completed a Web-based screener to determine eligibility; eligible and interested individuals provided consent online and completed the Web-based survey. Surveys were self-administered and could be completed at the place and on the device of the participant’s choosing (eg, mobile in a car, tablet at home, or computer at the CARE2HOPE storefront). Toward the end of the survey, participants were asked if they were interested in referring people to be screened for the study. Participants who agreed to help recruit were emailed or texted electronic recruitment coupons with a unique identifier that they could forward to others. Participants received US $10 for each of the first 3 eligible people they referred. Referred individuals, in turn, completed the Web-based screener, and eligible and consenting individuals took part in the Web-based survey and were given the opportunity to recruit others. Participants received US $30 for completing the survey.

As eligibility was ascertained online, we created an intensive Web-based screener to reduce fraud. This screening process included a quiz on the county the individual reported living in, and a quiz on the dose, appearance, and cost of the opioid they reported using most frequently in the past 30 days.

### Measures

Measures analyzed here included sociodemographic items (eg, age and gender); activity space pin-drop mapping items; and technical problems encountered during the survey.

#### Mapping Activity Space Locations

Before launching the survey, we conducted cognitive interviews with 4 young adults who met the survey’s eligibility criteria. These individuals completed the pin-drop mapping items in the presence of study staff and shared insights into how to address future participants’ concerns on disclosing the locations of highly sensitive and sometimes illegal behaviors and how to clarify mapping instructions. Suggestions (eg, highlight data confidentiality) were incorporated into the final survey’s mapping items.

**Figure 1:**
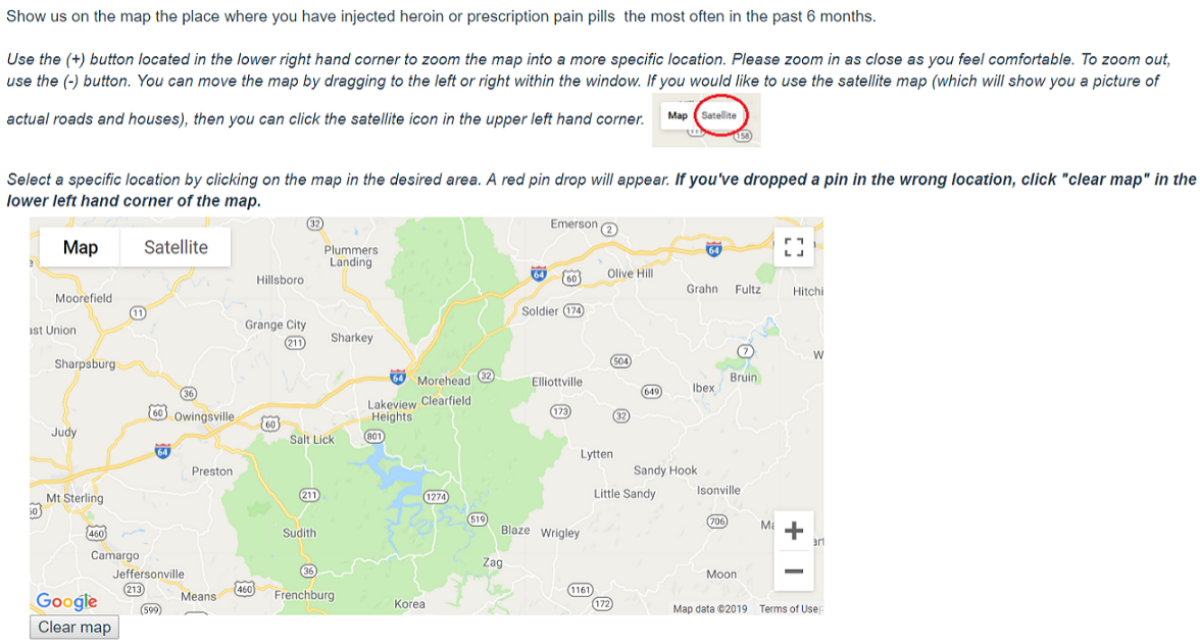
Survey item to gather pin-drop data on drug use location.

A secure digital map for each item was embedded in the Web-based survey, and participants were instructed to *drop a pin* on each map as close to the correct location as they felt comfortable (Figure 1). We created mapping items to capture 6 locations for each participant:

- Pin-drop home location: “Show us on the map where you have slept most in the past 6 months.”
- Pin-drop injection location: “Show us on the map the place where you have injected heroin or prescription pain pills the most often in the past 6 months.”
- Pin-drop noninjection opioid use location: “Show us on the map the place where you have taken heroin or prescription pain pills to get high WITHOUT INJECTING THEM the most often in the past 6 months.”
- Pin-drop transactional sex location: “Show us on the map where you have had VAGINAL or ANAL sex with someone to get drugs, money, housing or other resources most often in the past 6 months.”
- Pin-drop sex location (nontransactional): “Show us on the map where you have had vaginal or anal sex that was NOT in exchange for drugs, money, housing, or other resources most often in the past 6 months.”
- Pin-drop survey completion location: “Show us on the map where you are now.”

The consent form noted that individuals could skip items without jeopardizing their incentive.

During the analysis phase, we merged the items querying opioid injection location and smoking or snorting location into a single *drug use location* variable; participants who shared data on both were assigned to their injection location. Due to a coding error in the survey, individuals who reported only oral ingestion were not asked the noninjection drug use location item (N=45).

Each mapping item contained instructions about how to navigate the map, including how to zoom closer and remove an incorrectly dropped pin. All maps were centered in the county that contained the largest city in the study area.

The location where participants slept most was queried first (*pin-drop home location*). Every subsequent mapping item first asked whether the location was the same as the person’s home. Participants responding affirmatively skipped that mapping item, and their home location was used as the response, assessed using either the *pin-drop home location* or, if that was missing, the street address they entered into the screener for the place where they had *slept most in the past 6 months*. The survey was programmed so that participants skipped mapping items for behaviors in which they had not engaged in the past 6 months (eg, participants who had not engaged in transactional sex in the past 6 months were not asked to map the location of this behavior). All pins were automatically geocoded to their latitude and longitude in SurveyGizmo.

#### Measuring Precision

We assessed the precision of pin-dropped locations in 2 ways:

1. Using Google Maps, we manually assessed whether or not each pin-drop location was located within 100 m of a structure. Unpublished qualitative data from young adults who use opioids to get high and live in these counties suggested that, while they may use drugs or have sex outside at times, they greatly prefer to engage in these behaviors inside (eg, their home or their partner’s home). In this rural area with county-level population densities ranging from 31 to 84 people per square mile, the vast majority of the surface area does not have structures; randomly dropped pins would thus not be near a structure.
2. We calculated the Euclidean distance (ie, as the crow flies) between the pin-drop home location and the street address (street-address home location) where participants reported sleeping most in the past 6 months, an item queried in the screener. Street addresses were geocoded to their latitude and longitude location (96% match).

#### Assessing Technical Challenges

Participants answered questions about technical problems experienced while completing the survey, including while dropping pins on the maps. Technical problems queried included yes or no items on frozen screens, internet connectivity, and problems saving answers. Questions specific to the mapping items included the following: maps were slow to load, maps would not zoom in or out, and trouble dropping a pin on the map in the correct place.

### Analysis

We used several methods to detect and eliminate fraud [17]. First, the survey experienced an onslaught of entries during two 48-hour periods from individuals who reported living in the same house. A scan of the house suggested that it could not be home to so many people, and so we excluded all participants who completed the survey during those periods who listed that house as their address (N=49 entries excluded). Next, we applied components of a fraud-detection protocol created by Ballard and colleagues to identify fraud in Web-based surveys [17]. We investigated repeated names and dates of birth across entries and inconsistencies in names within entries. This excluded another 12 entries. Finally, we eliminated individuals who completed the survey implausibly rapidly (<10 min for people who injected; <8 min for others), excluding another 6 entries. The final sample included 151 participants.

Descriptive statistics were used to explore distributions of variables across this sample of 151 participants. Chi-square, independent t-tests for age and monthly income, and Fisher exact tests were used to detect group differences in the likelihood of responding to mapping items, and in the distance between the address-based home location and pin-drop home location (*precision analysis*). Variables of interest included a range of sociodemographic characteristics (age, educational attainment, homelessness status, car access, parenting status, and whether the participant lived in the county on which the maps were centered); injection drug use status; criminal justice involvement; and recruitment method (eg, peer referral, CARE2HOPE staff). The number of people who reported transactional sex (N=55) was too small to support analyses of the correlates of missingness and precision.

We limited the sample for the precision analyses in 2 ways:

1. Each participant’s street-address home location was queried in the screener and at several points in the survey. To help ensure that the street-address home location could be considered the gold standard against which pin-drop home locations were compared, we restricted the sample to participants who reported the same street address on the screener and on at least one survey item capturing home address (N=96).
2. In a corollary analysis, we further reduced the sample to participants who reported no technical difficulties completing the survey (N=80) to try to capture threats to precision above and beyond these technical difficulties.

We used ArcGIS 10.6 Network Analyst to calculate distances and activity spaces [18]. Statistical analyses were conducted in SAS version 14.2 [19].

### Ethics

The Emory institutional review board (IRB) approved study protocols, and the University of Kentucky’s IRB deferred to this Board. A Certificate of Confidentiality was secured to protect data from subpoena.

## Results

### Sample Overview

A total of 151 valid surveys were completed. On average, survey participants were aged 28.9 years (standard deviation [SD] 4.1), and 61.6% (93/151) were men (Table 1). Consistent with the racial or ethnic composition of the area, 96.7% (146/151) of participants identified as white, and 98.7% (150/151) identified as non-Hispanic. The sample was deeply impoverished: the median monthly income was US $300 (interquartile range: US $664), and almost half (45.0% [68/151]) were currently homeless (ie, living on the street, or in a car, park, abandoned building, or shelter). Two-thirds of participants were recruited via CARE2HOPE staff contact; 27.8% (42/151) was recruited via peer referral. Over 70% (109/151) of the sample had used heroin in the past 6 months; 51.7% (78/151) reported using prescription pain pills to get high in the past 6 months. Two-thirds of the sample reported injecting drugs in the past 6 months.

**Table 1:** Participant characteristics and difficulties encountered while completing an online survey, for a sample of young adult residents of rural Appalachian Kentucky who used opioids to get high (N=151).

Participant characteristic		Values^a^
Age (years), mean (SD)		28.9 (4.1)
**Gender, n (%)**		
	Man	93 (61.6)
	Woman	58 (38.4)
**Race, n (%)**		
	White	146 (96.7)
	Black or African American	4 (2.7)
	Other	1 (0.7)
Hispanic/Latino ethnicity, n (%)		1 (0.7)
Homeless, n (%)		68 (45.0)
**Educational attainment, n (%)**		
	Less than high school	46 (30.5)
	High-school graduate	59 (39.1)
	Some college or associate’s degree	40 (26.5)
	College graduate or higher	3 (2.0)
**Employment status, n (%)**		
	Full-time	27 (17.1)
	Part-time	28 (17.7)
	Other	103 (65.2)
Monthly income (US $), mean (SD)		300 (664)
Usually drive myself where I need to go, n (%)		69 (45.7)
Caregiver to a child, n (%)		29 (19.2)
History of arrest, n (%)		60 (39.7)
Injected drugs to get high (past 6 months), n (%)		100 (66.2)
**Primary drugs used (multiple responses possible), n (%)**		
	Heroin	109 (72.2)
	Methamphetamines	89 (58.9)
	Buprenorphine or methadone	92 (60.9)
	Opioid prescription pain pills	78 (51.7)
	Gabapentin or Neurontin	60 (39.7)
	Prescription sedatives/tranquilizers	40 (26.5)
	Crack or cocaine	34 (22.5)
Live in the most populous county (ie, where maps centered), n (%)		118 (78.2)
**Recruitment method, n (%)**		
	Staff contact	100 (66.2)
	Peer referral	42 (27.8)
	Other	9 (6.0)
**Device used to complete the survey, n (%)**		
	Mobile phone	72 (48.3)
	Computer	43 (28.9)
	Tablet	16 (10.7)
	Other	18 (12.1)
**Technical challenges experienced while completing the survey (participants could select >1 response), n (%)**		
	None	119 (78.8)
	Maps were slow to load	10 (6.6)
	Trouble dropping pin in the correct place	10 (6.6)
	Maps would not zoom in or out	8 (5.3)
	Lost internet connection	4 (2.2)
	Other	10 (6.6)

^a^Some percentages do not sum to 100% because of some individuals refused to answer select items.

### Technical Challenges While Completing the Survey

More than three-fourths (78.8% [119/151]) of the sample reported that they had had no problems completing the Web-based survey (Table 1). Of those who reported a problem, the most commonly reported problems were that the maps were slow to load (6.6% [10/151]); that it was difficult to drop the pin in the correct location (6.6% [10/151]); and that it was hard to use the *zoom* feature on the maps (ie, zoom in or out; 5.3% [8/151]).

### Activity Space Missingness Analysis

**Figure 2:**
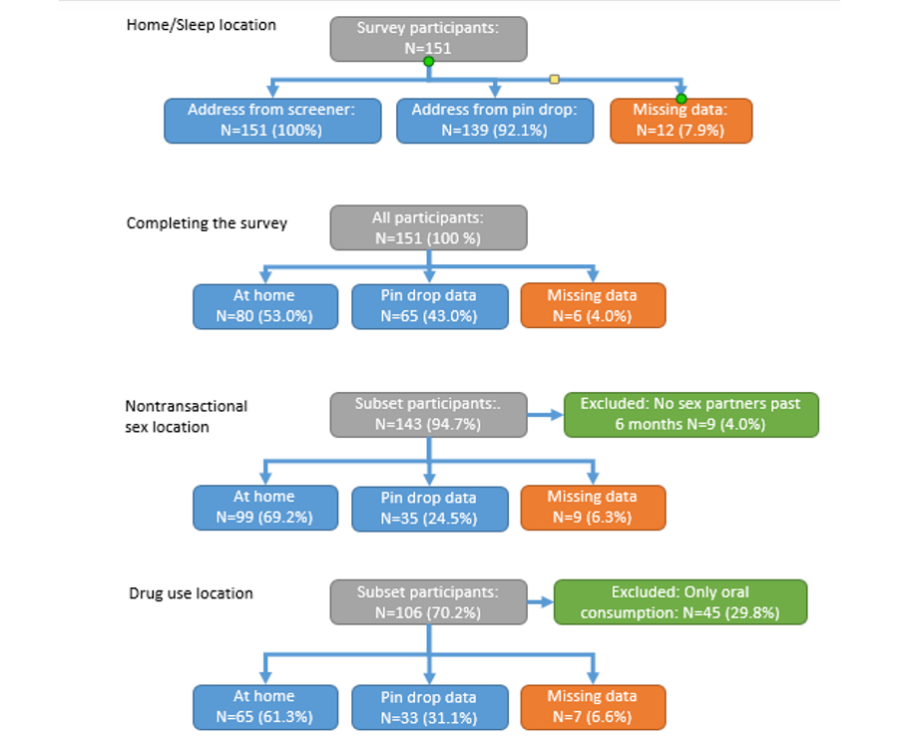
Location data missingness flowchart.

Of the 151 participants, 88.7% (134/151) dropped pins for all locations they were asked to locate. All 151 participants were asked to drop a pin where they had slept most in the past 6 months, and 92.1% (139/151) dropped a pin in answer to this question (Figure 2; Table 2). Participants who worked full time were less likely to complete this item than part-time workers or others (78% [18/23], 92% [22/24], and 95% [89/94], respectively), as were participants who did not live in the county where the map was centered (76% [25/33] vs 96.6% [114/118]). Participants recruited by CARE2HOPE staff were more likely to drop a pin at their home than participants recruited by peers or others (96% [96/100], 83% [35/42], and 89% [8/9], respectively). There was a borderline statistically significant relationship (*P*=.07) suggesting that participants who had good car access were less likely to drop a pin at their home than others (87% [60/69] vs 96% [79/82]).

All 151 individuals were asked to map their current location (ie, where they were completing the survey), and 96.0% (145/151) reported this location either by dropping a pin or by noting it was the same location as their home (Table 2). There was a borderline statistically significant relationship (*P*=.09) suggesting that people who were recruited by CARE2HOPE staff members were more likely to report this location (98% [98/100] for staff referral; 93% [39/42] for peer referral; 89% [8/9] for other referral methods). Missingness on current location did not vary systematically by any other participant characteristics, including age, gender, homeless status, or educational attainment.

**Table 2:** Patterns of completion for pin-drop mapping items of home location and current location, completed by a sample of young adults in rural Appalachian Kentucky who use opioids to get high (N=151). Some percentages do not sum to 100% because of some individuals refused to answer select items.

Participant characteristics		Home		Current location	
		Dropped pin	*P* value	Dropped pin	*P* value
**Age (years), mean (SD)**			.85		.44
	Dropped pin	28.9 (4.1)		28.8 (4.1)	
	Did not drop pin	28.7 (4.3)		30.2 (3.7)	
**Gender, n (%)**			.21		.68
	Man	88 (94.6)		90 (96.8)	
	Woman	51 (87.9)		55 (94.8)	
**Homeless, n (%)**			.35		.41
	Yes	61 (89.7)		64 (94.1)	
	No	77 (95.1)		79 (97.5)	
**High-school graduate, n (%)**			.75		.65
	No	43 (93.5)		44 (95.7)	
	Yes	93 (91.2)		99 (97.1)	
**Employment status, n (%)**			.04		.31
	Full-time	18 (78.3)		21 (91.3)	
	Part-time	22 (91.7)		23 (95.8)	
	Other	89 (94.7)		91 (96.8)	
**Monthly income (US $), median (IQR)**			.16		.94
	Dropped pin	650 (300)		300 (664)	
	Did not drop pin	100 (593)		300 (600)	
**Usually drive myself, n (%)**			.07		.41
	Yes	60 (87.0)		65 (94.2)	
	No	79 (96.3)		80 (97.6)	
**Caregiver, n (%)**			.39		.99
	Yes	26 (89.7)		28 (96.6)	
	No	105 (94.6)		108 (97.3)	
**History of arrest, n (%)**			.63		.63
	Yes	58 (96.7)		59 (98.3)	
	No	68 (89.5)		73 (96.1)	
**Live in county where map centered, n (%)**			.001		.12
	Yes	114 (96.6)		115 (97.5)	
	No	25 (75.8)		30 (90.9)	
**Injected (past 6 months), n (%)**			.99		.99
	Yes	94 (94.0)		98 (98.0)	
	No	41 (91.1)		45 (100.0)	
**Recruitment method, n (%)**			.03		.09
	Staff contact	96 (96.0)		98 (98.0)	
	Peer referral	35 (83.3)		39 (92.9)	
	Other	8 (88.9)		8 (88.9)	
**Device used, n (%)**			.77		.34
	Mobile phone	66 (91.7)		69 (95.8)	
	Computer	40 (83.0)		43 (100.0)	
	Tablet	15 (93.8)		15 (93.8)	
	Other	18 (13.0)		18 (100.0)	
**Reported a technical problem with this survey, n (%)**			.99		.34
	Yes	28 (93.3)		28 (93.3)	
	No	111 (91.7)		117 (96.7)	

Of the 143 participants who had been sexually active in the past 6 months, 93.7% (134/143) marked the place where they had (nontransactional) sex most often in the past 6 months, either by dropping a pin or by reporting that the location was the same location as their home (Table 3). People recruited by CARE2HOPE staff members or through *other methods* were more likely to report this location than other participants (97% [89/92] for staff referral; 100% [8/8] for other means; and 86% [36/42] for peer referral), as were participants who reported living in the county where the map was centered (96.5% [110/114] vs 83% [24/29]). Missingness on this item did not vary by other participant characteristics, though there were borderline statistically significant relationships (.05≤*P≤*.10) suggesting that women (*P*=.09) and housed (*P*=.08) individuals were more likely to report this location and that people who were employed full time were less likely to do so (*P=*.08).

Of the 106 participants who were asked the drug use location item, 93.4% (99/106) either dropped a pin in this location or reported that it was the same location as their home. Missingness varied by the device participants used to complete the survey, with higher completion rates for this item among participants using computers or mobile phones (100% [28/28] and 96% [52/54], respectively) than tablets and other devices (78% [7/9] and 85% [11/13], respectively). Participants who lived in the county the map was centered on were more likely to complete this item (96% [78/81] vs 84% [21/25]). Missingness on this item did not vary systematically by other participant characteristics.

**Table 3:** Patterns of completion for pin-drop mapping items of sex location and drug use location completed by a sample of young adults in rural Appalachian Kentucky who use opioids to get high (N=151). Some percentages do not sum to 100% because of some individuals refused to answer select items.

Participant characteristics		Sex location		Drug use location	
		Dropped pin	*P* value	Dropped pin	*P* value
**Age (years), mean (SD)**			.87		.83
	Dropped pin	28.8 (4.1)		29.1 (4.3)	
	Did not drop pin	29.0 (4.0)		29.4 (3.6)	
**Gender, n (%)**			.09		.60
	Man	78 (90.7)		61 (92.4)	
	Woman	56 (98.3)		38 (95.0)	
**Homeless, n (%)**			.08		.70
	Yes	57 (89.1)		44 (91.7)	
	No	75 (97.4)		54 (94.7)	
**High-school graduate, n (%)**			.99		.19
	No	40 (93.0)		25 (100.0)	
	Yes	91 (93.8)		72 (91.1)	
**Employment status, n (%)**			.08		.36
	Full-time	18 (81.8)		14 (87.5)	
	Part-time	22 (95.7)		18 (100.0)	
	Other	84 (95.5)		59 (92.2)	
**Monthly income (US $), median (IQR)**			.28		.20
	Dropped pin	300 (635)		300 (600)	
	Did not drop pin	100 (400)		2.5 (800)	
**Usually drive myself, n (%)**			.74		.71
	Yes	63 (92.7)		48 (92.3)	
	No	71 (94.7)		51 (94.4)	
**Caregiver, n (%)**			.99		.57
	Yes	27 (96.4)		22 (100.0)	
	No	98 (94.2)		72 (94.7)	
**History of arrest, n (%)**			.70		.69
	Yes	53 (96.4)		42 (95.5)	
	No	68 (93.2)		49 (92.5)	
**Live in county where map centered, n (%)**			.02		.05
	Yes	110 (96.5)		78 (96.3)	
	No	24 (82.8)		21 (84)	
**Injected (past 6 months), n (%)**			.38		.22
	Yes	91 (96.8)		77 (97.5)	
	No	40 (93.0)		21 (91.3)	
**Recruitment method, n (%)**			.05		.17
	Staff contact	89 (96.7)		70 (95.9)	
	Peer referral	36 (85.7)		23 (88.5)	
	Other	9 (100.0)		6 (85.7)	
**Device used, n (%)**			.59		.03
	Mobile phone	64 (91.4)		52 (96.3)	
	Computer	39 (97.5)		28 (100.0)	
	Tablet	14 (100.0)		7 (77.8)	
	Other	16 (94.1)		11 (84.6)	
**Reported a technical problem with survey, n (%)**			.99		.64
	Yes	21 (91.3)		27 (93.1)	
	No	78 (94.0)		107 (93.9)	

### Precision

Two-thirds of the pin-drop sex and drug use locations were within 100 m of a structure, as were 92.1% (128/139) of pin-drop home locations. As noted, we restricted the precision analysis of the distance between pin-drop home location and street-address home location to participants whose screener-reported home address was identical to the home address they reported on at least one other survey item (N=96). The median Euclidean distance between the pin-drop home location and the street-address home location was 2.0 miles (25th percentile: 0.8 miles; 75th percentile: 5.5 miles). The distance between these 2 home locations varied by educational attainment, recruitment method, injection status, and experiencing technical difficulties with the survey (Table 4). Specifically, the median distance was smaller for high-school graduates (1.6 miles for high-school graduates vs 2.5 miles for people who did not graduate high school); for people recruited by staff (1.8 miles for staff recruits; 4.0 miles for peer recruits; 11.3 miles for other recruitment methods); for people who injected drugs (1.9 miles for people who currently injected vs 3.6 miles for others); and for participants reporting no technical difficulties with the Web-based survey (1.8 miles vs 5.6 miles). There was a borderline statistically significant (.05<*P*<.09) relationship suggesting that participants completing the mapping items on tablets had a longer median distance (1.9 miles if completed on a computer; 2.1 miles on a mobile phone; 6.8 miles on a tablet).

Analyses that excluded participants reporting technical difficulties generated medians and 25th percentiles that were similar to those generated by the full sample (Table 4; N=80), but the values of the 75th percentiles were slightly lower than in the full sample. For example, the 75th percentile of the distance measure for participants who usually drove themselves was 2.8 miles in the reduced sample, whereas it was 6.5 miles in the full sample; it was 4.4 miles for women in the restricted sample, whereas it was 6.3 miles for women in the full sample. Individual-level correlates were similar across both samples.

**Table 4:** Distributions of the number of miles between pin-drop home locations and street-address home locations for a sample of young adults living in rural Appalachian Kentucky who use opioids to get high.

Characteristics		Sample (N=96)				Sample restricted to participants who reported no technical problems completing the survey (N=80)			
		Percentile			*P* value	Percentile			*P* value
		25th	50th	75th		25th	50th	75th	
Age (years)		27.0	29.0	32.0	.85	27.0	29.5	32	.88
**Gender**					.57				.52
	Man	1.1	2.1	5.4		0.8	2.0	4.6	
	Woman	0.5	1.6	6.3		0.5	1.6	4.4	
**Homeless**					.56				.46
	Yes	1.2	2.2	5.7		0.8	2.1	4.6	
	No	0.6	2.0	5.3		0.7	1.6	2.8	
**High-school graduate**					.03				.03
	No	1.7	2.5	6.9		1.5	2.2	5.7	
	Yes	0.5	1.6	4.9		0.5	1.6	4.1	
**Employment status**					.23				.34
	Full-time	0.6	3.7	5.7		0.7	3.9	5.7	
	Part-time	1.8	3.6	7.3		2.1	2.1	5.4	
	Other	0.8	1.8	4.6		1.6	1.6	2.9	
Monthly income (US $)		100.0	300.0	661.5	.57	10.0	275.0	60.0	.71
**Usually drive myself**					.93				.46
	Yes	0.8	2.1	4.8		0.8	2.1	4.6	
	No	0.9	2.0	6.5		0.7	1.6	2.8	
**Caregiver**					.48				.58
	Yes	0.4	1.9	5.3		0.4	1.3	4.6	
	No	1.1	2.0	5.0		1.1	1.8	3.7	
**History of arrest**					.75				.72
	Yes	0.8	2.0	5.1		0.6	1.7	4.3	
	No	0.8	2.1	5.3		0.9	3.3	3.3	
**Injected (past 6 months)**					.047				.07
	Yes	0.8	1.9	4.1		0.6	1.8	2.8	
	No	0.8	3.6	14.1		0.8	2.5	16.4	
**Live in county where map centered**					.48				.41
	Yes	0.8	2.0	4.8		0.7	1.8	4.1	
	No	0.8	6.9	12.6		0.8	2.8	12.6	
**Recruitment method**					.03				.02
	Staff contact	0.7	1.8	4.5		0.6	1.6	2.9	
	Peer referral	1.3	4.0	14.2		1.4	4.0	17.7	
	Other	2.0	11.3	31.2		2.0	2.4	2.2	
**Device used**					.07				.26
	Mobile phone	0.6	2.1	5.4		0.6	2.1	5.4	
	Computer	1.2	1.9	3.2		1.2	1.7	2.9	
	Tablet	3.4	6.8	35.0		0.6	4.6	36.2	
	Other	0.7	1.6	3.4		0.5	1.3	2.0	
**Reported technical problem with survey**					.04				—^a^
	Yes	1.6	5.6	7.8		—	—	—	
	No	0.8	1.8	4.5		—	—	—	

^a^Not applicable.

## Discussion

### Principal Findings

In this sample of rural young adults who use opioids to get high, we find that self-administered pin-drop maps hold some promise as a tool to capture information on risk-related activity spaces. Response rates to mapping items were high, and most pin-drops were near structures, but the distance between pin-drop home locations and street-address home locations might be unacceptably large for some subpopulations and analytic purposes. We describe and contextualize these results and offer recommendations to strengthen future research.

Though participants were told that they could still receive their incentive if they skipped items, almost 90% of participants dropped pins for all the locations for which they were eligible, and item-specific response rates exceeded 92%. These results suggest that analyses of mapping items in Web-based surveys with rural young adults who use drugs should be as adequately powered as analyses of other survey items.

These high response rates may be attributed, in part, to the prominence of the study’s federal Certificate of Confidentiality in the survey. These Certificates “…protect the privacy of research subjects by prohibiting disclosure of identifiable, sensitive research information to anyone not connected to the research except when the subject consents or in a few other specific situations” [20]. Past research with rural people who use drugs suggests that they view these certificates as *trust agreements* that signal that the data will be kept private [21]. We ensured that the Certificate figured prominently in the survey. In addition to describing the Certificate in the informed consent, the survey contained repeated reminders about this certificate, and we strategically placed these reminders before sections querying sensitive and illegal behavior.

Past research has found that willingness to respond to location items varies by sociodemographic characteristics, a pattern that may introduce systematic bias [14]. Here, we found borderline statistically significant relationships suggesting that men and homeless individuals were less likely to report the location where they had sex. As Vaughan and colleagues have found [14], reporting this location may be particularly sensitive because it may reveal the identity of an intimately known person, if sex occurs at a primary partner’s home. Homeless individuals may be especially likely to have sex at another person’s home; possibly, heterosexual men were protective of female partners if they tended to have sex at this partner’s home. Individuals who were employed full time were less likely to drop pins at their sex and home locations, perhaps because they were especially fearful of losing their job if survey data became public.

We also found that response rates varied by whether individuals were recruited by staff members, and whether the individual lived in the county where the maps were centered. Past qualitative research conducted in rural Kentucky with people who use drugs provides possible explanations for the recruitment findings. Participants in that qualitative study reported that they would be more likely to disclose location data if they had a strong rapport with interview staff [21]. Our recruitment cookouts were designed to provide opportunities for potential participants to develop rapport with study staff. Cookouts were low-threshold community events in which local residents could choose if and when to approach study staff; if they chose to visit the cookout, they could sit, dine, and chat with study staff and other community residents before deciding whether to self-administer the Web-based screener. We also recruited at the CARE2HOPE project’s storefront; CARE2HOPE is conducted with local people who use drugs and staffed by team members who clearly communicate respect for the dignity of people who use drugs. The rapport developed with study staff at the cookouts and in the CARE2HOPE storefront may have increased participants’ willingness to report sensitive data. In contrast, when peers recruited participants into the study, they did so via digital peer referral (ie, invitations sent by text messages and emails). Individuals recruited through this method may have had multiple concerns, including about the authenticity of the texted or emailed coupons, and about the trustworthiness of faceless researchers. Overall, however, there were few differences in response rates by participant-level characteristics, including age, income, homeless status, or educational attainment, indicating that responses to these items should not be systematically biased by these participant characteristics.

Maps were centered on the county with the largest population size, and people living outside this county were more likely to have missing data on their home location and their sex and drug use locations. We attribute this missingness pattern to problems navigating the maps across the large 5-county surface area (1378 square miles).

We analyzed the precision of pin-drop home locations in 2 ways: examining their proximity to structures and assessing their distance from self-reported street addresses. In a set of rural counties where the vast majority of the 1378 square mile surface area is empty of structures, a high percentage (92%) of participants’ pin-drop home locations were within 100 m of a structure. These pin-drops were, however, a median of 2 miles from their reported home address. There is no gold standard for an acceptable magnitude of error for pin-drops, and this magnitude may vary by purpose. Errors of ≤2 miles between the pin-drop location and the actual location may be acceptable for analyses designed to identify optimal locations of health and social service sites in rural areas, which may necessarily be many miles from most potential clients’ homes. Errors of this magnitude, however, may be unacceptably large when investigators are characterizing exposures whose influence depends on close proximity (eg, abandoned buildings). A median error of 2 miles may indicate that participants had trouble navigating to the precise point where their home was located or might have intentionally masked their home location. Supporting the latter interpretation and perhaps for the reasons offered above to explain correlations of missingness and staff recruitment, distances were shorter for staff-recruited participants. Supporting the former interpretation, participants experiencing no technical difficulties or participants who graduated from high school had smaller mapping errors. Rerunning the distance analysis on the subset of participants who reported no technical difficulties resulted in somewhat lower 75th percentile values, suggesting that there were fewer random pin-drops for this subgroup. Although people who injected drugs had shorter distances between their pin-drop and address-based home locations, this may have been an artifact of recruitment method: people who injected were disproportionately likely to have been recruited by staff (*P*=.008).

We found that 64% of pin-drop sex and drug use locations were within 100 m of a structure, a figure that is lower than the 92% found for pin-drop home locations. We conducted post hoc analyses of survey items querying the specific settings where participants engage in risk behaviors, and these post hoc analyses indicate that many participants reported having sex and using drugs outside in the past 6 months, though our unpublished qualitative data had suggested that they preferred to engage in these behaviors inside. For example, 26% reported having sex in a car; 7% reported having sex in a cemetery; and 11% reported having sex in another outdoor location. More than half of the sample (55.4%) reported injecting drugs in a car in the past 6 months; approximately one-fourth reported injecting in a cemetery and one-third reported injecting in another outdoor location. Many participants thus appear to use drugs and have sex in locations that may in fact have been far from a structure.

Few participants reported difficulties completing pin-drop items: 79% reported no technical problems with the Web-based survey whatsoever, and less than 7% of participants endorsed for any mapping-specific difficulties. We note, though, that other rural areas and other populations (eg, older rural adults) may have a poor internet service. For these populations, Web-based mapping items might load slowly or might not load at all; in addition, members of these populations may be unfamiliar with Web-based maps and thus less able to successfully navigate through them.

### Limitations

Analyses were conducted on a convenience sample and thus might not be representative of the underlying population of young adults living in the 5-county area who use opioids to get high. Findings may not generalize to older people who use drugs, who may have poorer access to the internet and may be less able to navigate pin-drop maps because of poorer digital literacy [22]. Our measures of activity locations did not encompass the full range of activities in which this population engages (eg, locations where they socialized, shopped, and worked). Our measure did, however, capture locations that people who use drugs may be especially reluctant to disclose. In addition, we did not query the street address for drug use and sex locations and thus could not compare pin-drop and street-address locations for these activities. As noted, however, many participants used drugs and had sex outside in places without street addresses. Same-source bias is a possible limitation for our effort to assess the precision of pin-drop home locations using street address data: individuals may have simply reported someone else’s home address and dropped a pin at that location. A high percentage (45%) of participants in this sample reported being homeless. Homeless populations are heterogeneous and may vary in ways that influence the geographic locations where they sleep and engage in risk behaviors, the characteristics of these places, and their willingness to report geospatial information. Future research should develop a larger sample of homeless individuals that is sufficiently powered to explore these variations.

### Conclusions and Recommendations

Research methods must keep pace with expanding drug-related crises in rural areas. As public health theories and interventions increasingly recognize the roles of place characteristics in shaping disease and health service use [5-7], researchers have started using pin-drop maps to capture activity spaces among urban populations [14,23]. This analysis suggests that, with some methodologic improvements, pin-drop maps may also be a promising method of collecting data on risk-related activity spaces among rural young adults who use drugs, a population that is understudied and yet at high risk of a host of adverse health outcomes. Specific recommendations to strengthen pin-drop methods to define activity spaces in this population include the following:

- Clearly describing certificates of confidentiality and strategically highlighting it at various points in the survey
- Recruiting participants via staff who are trained to treat people who use drugs with dignity
- Centering maps on the county where the participant lives. This may require that survey programmers have significant technical skills
- Offering the option of completing mapping items with a staff member, if participants have not graduated from high school or encounter technical difficulties
- Gathering metadata on the number of attempts needed to drop each pin (or the number of seconds needed to drop each pin), and whether this number correlates with precision measures
- Including survey items querying participant concerns about providing specific location data for each location type
- Gathering metadata or self-report data on the extent to which participants zoom in or out on each map.

Future research should also expand the range of locations queried (eg, places where people who use drugs socialize, work, shop, worship, and seek health and social services) and populations sampled (eg, older rural people who use drugs) and continue to assess the extent to which missingness and precision vary across and within these new places and populations. As this novel line of inquiry advances into these new places and populations, conducting formative research (eg, cognitive interviews, as was done here) will be vital to address concerns about human subjects’ protections, and to identify—and develop strategies to overcome whenever possible—barriers and potential biases in responses.

## Abbreviations

ARC: Appalachian Regional Commission
CDC: Centers for Disease Control and Prevention
GPS: global positioning system
HCV: hepatitis C virus
IRB: institutional review board
PIs: principal investigators
SAMHSA: Substance Abuse and Mental Health Services Administration
